# Impact of lifestyle factors post-infectious mononucleosis on multiple sclerosis risk

**DOI:** 10.1007/s10654-025-01212-1

**Published:** 2025-03-04

**Authors:** Eva Johansson, Tomas Olsson, Lars Alfredsson, Anna Karin Hedström

**Affiliations:** 1 Department of Clinical Neuroscience, Karolinska Institutet, Stockholm, Sweden; 2 Center for Molecular Medicine, Karolinska Institutet, Stockholm, Sweden; 3 Centre for Occupational and Environmental Medicine, Region Stockholm, Stockholm, Sweden; 4 Institute of Environmental Medicine, Karolinska Institutet, Stockholm, Sweden

## Abstract

**Background:**

Accumulating evidence suggest that Epstein-Barr virus (EBV) is crucial in the development of multiple sclerosis (MS), with inadequate infection control possibly contributing to disease onset. Past infectious mononucleosis (IM) has been found to interact with smoking, obesity, and sun exposure. We aimed to investigate potential interactions between a history of IM and the following risk factors for MS: passive smoking, alcohol consumption, fish consumption, vitamin D status, adolescent sleep duration and sleep quality.

**Methods:**

We analyzed data from a Swedish population-based case-control study (3128 cases and 5986 controls). Subjects were categorized based on IM status and each exposure variable and compared regarding MS risk by calculating odds ratios (OR) with 95% confidence intervals (CI) using logistic regression models. Additive interaction between aspects of IM status and each exposure was assessed by calculating the attributable proportion due to interaction (AP) with 95% CI.

**Results:**

The OR of developing MS among those who reported a history of IM was 1.86 (95% CI 1.63–2.12), compared with those who had not suffered from IM. We observed synergistic effects between a history of IM and each exposure variable with respect to risk of MS, with significant APs ranging between 0.20 and 0.35.

**Conclusions:**

The concept of EBV infection as a crucial factor for MS gains further support from our findings suggesting that MS risk factors synergize with a history of IM in disease development. Targeting modifiable MS risk factors that impede effective immune regulation of the virus holds promise for preventive interventions.

## Introduction

Accumulating evidence suggests that infection with Epstein-Barr virus (EBV) is a prerequisite for the onset of multiple sclerosis (MS), an inflammatory and neurodegenerative disease of the central nervous system. Nearly all patients with MS are EBV-seropositive at the onset of the disease [1], whereas the risk of MS in seronegative individuals remains exceptionally low [2]. Nonetheless, the majority of seropositive individuals will never develop MS, hinting at the significance of adequate viral control in the disease’s pathogenesis. It is plausible that inadequate control of the virus may be another prerequisite for disease onset.

According to Rothman’s sufficient cause model, the onset of a complex disease, such as MS, results from the cumulative effects of multiple risk factors reaching a threshold level of causality, thereby initiating the disease progress (3–4). If EBV serves as a necessary but not sufficient factor for MS, it would need to combine with other risk factors to cause the disease. This means that EBV interacts with other risk factors, contributing to the overall cause of MS.

While ideally, additive interaction would be tested between EBV seropositivity and other risk factors for MS, the near-universal EBV seropositivity among MS patients presents a challenge in examining such interactions. Consequently, our focus shifts to exploring interactions between a history of infectious mononucleosis (IM) and risk factors for MS. IM has consistently been associated with increased risk of MS [5] and may serve as a proxy for insufficient viral control, thus warranting investigation into its potential interactions with other MS risk factors.

Several studies have demonstrated the presence of interactions between aspects of EBV infection and established risk factors for MS, including genetic predisposition [6–8], smoking (9–10), obesity [11], and low sun exposure [12]. In the present study, we selected risk factors based on their potential to influence MS risk through biologically plausible pathways, particularly those relevant to immune regulation, neuroinflammation, and the interplay with EBV infection. These include passive smoking, alcohol consumption, fish consumption, vitamin D status, adolescent sleep duration and sleep quality. Although the evidence supporting these factors varies, their inclusion allows for a comprehensive exploration of potential interactions with a history of IM, providing valuable insights into the multifactorial nature of MS pathogenesis.

## Methods

### Design and study population

We utilized data from a population-based nation-wide case-control study, the Epidemiological Investigation of Multiple Sclerosis (EIMS), which involved individuals aged 16–70 years from the Swedish general population. Incident cases of MS were identified from neurology units in hospitals and private clinics, with diagnoses confirmed by local neurologists using the McDonald criteria by local neurologists (13–14). For each case, we randomly selected two controls from the national population register (in close connection in time to the inclusion of the case in question), matched on age (within 5-year age groups), sex, and residential area. Detailed information about the structure and methodology of the study has been described elsewhere [11].

All participants completed a standardized questionnaire, providing information on environmental exposures and lifestyle factors. They were also asked to donate blood samples for genetic and serologic analysis. Over the study period April 2005 to December 2021, we obtained questionnaires from 3596 cases and 6917 controls, resulting in response rates of 93% and 73%, respectively. Any incomplete responses were supplemented by phone or mail. In November 2013, we administered additional questions, including inquiries about sleep habits, to all participants. These questions were subsequently incorporated into the standard EIMS questionnaire. For the current study, participants who were unsure about their history of IM were excluded, resulting in a final sample of 3128 cases and 5986 controls.

The study was approved by the Regional Ethical Review Board at Karolinska Institute (2004/1–4:6) and conducted in accordance with the ethical standards outlined in the 1964 Declaration of Helsinki and its later amendments. Participants provided informed consent before participating in the study.

### Definition of exposures

To ensure sufficiently large sample sizes for interaction analyses, most exposure variables were dichotomized using the median value among controls as the threshold. The corresponding questions can be found in Supplementary Table 1.

#### Passive smoking

Participants were asked about their lifetime exposure to passive smoking, either at home or in the workplace. Passive smoking status was dichotomized into affirmative or negative responses.

#### Alcohol consumption

Participants provided information regarding their weekly alcohol intake at the time of diagnosis. One glass of alcohol was defined as 12 g of alcohol. We dichotomized alcohol consumption into low (no or low consumption) or high (moderate or high consumption). The cutoff for low alcohol consumption (< 50 g/week for females and < 100 g/week for males) was the same as that used by Statistics Sweden, a governmental agency responsible for official statistics.

#### Fish consumption

Participants were queried about their frequency of lean and fatty fish consumption over the past five years. Responses were rated on a four-point scale ranging from never/seldom to daily. An index was created by summing the responses, resulting in values between 2 (lowest exposure) and 8 (highest exposure). Low fish consumption was defined as a value below the median among controls (< 5), while higher consumption was categorized as high fish consumption.

#### Vitamin D status

For participants enrolled between 2005 and 2009 (*n* = 2464), vitamin D status was assessed by measuring 25-hydroxy-vitamin D levels using a chemiluminescent immunoassay from Diasorin (Diasorin AB, Sundbyberg, Sweden) and a LIAISON^®^ instrument provided by Diasorin AB, which enabled equimolar measurement of both 25-hydroxy-vitamin D2 and D3. The analysis of vitamin D in the blood samples was conducted in a single batch. Vitamin D levels were categorized as low or high based on the median value among controls (63 nmol/l).

#### Adolescent sleep duration

Participants were requested to estimate their typical bedtime and wake-up time on work or school days across various age groups. They also provided details about their weekend or free day sleep schedule. Total sleep duration was calculated as the average nightly sleep duration over one week. Sleep duration was categorized as short or long based on the median sleep duration among controls (8.4 h per night).

#### Sleep quality

Subjects were asked to estimate the quality of their sleep during different age periods using a 5-grade scale (very bad, rather bad, neither good nor bad, rather good, very good). Sleep quality (15–19 years) was categorized into high (rather good or very good) or low (neither good nor bad, rather bad, very bad).

### Statistical analysis

Categorical variables were summarized using frequency and percentage. Continuous variables were summarized using mean and standard deviation (SD) or median and interquartile range (IQR) as appropriate.

The participants were categorized based on their IM status and each dichotomized exposure variable. The risk of MS was then assessed by computing odds ratios (OR) with corresponding 95% confidence intervals (CI) using logistic regression models. The reference group comprised individuals with low exposure levels to the investigated factors and no history of IM. The analysis regarding passive smoking was conducted in the overall sample and then further examined within the subgroup of never smokers.

Potential interactions on the additive scale, indicating departure from the additive effects, between a history of IM and each exposure variable were evaluated. This was accomplished by computing the attributable proportion due to interaction (AP) along with its corresponding 95% confidence intervals (CI). The AP represents the combined effect beyond the sum of the individual effects when two factors interact.

All analyses were adjusted for age, sex, residential area, ancestry, smoking, sun exposure, and adolescent body mass index (BMI). Ancestry was dichotomized into Nordic and non-Nordic origin. Smoking was categorized into current, past, or never smoking at index. We constructed a continuous variable for sun exposure, based on three questions regarding exposure to ultraviolet radiation where each answer alternative was given a number ranging from 1 (the lowest exposure) to 4 (the highest exposure). The numbers were added together into a sun exposure index and subjects were dichotomized into low or high sun exposure based on the median value among controls [23]. Adolescent body mass index was categorized into underweight, normal weight, overweight, and obesity, according to the cutoffs used by the World Health Organization. The analysis regarding vitamin D was further adjusted for month of blood sampling. We conducted a sensitivity analysis on alcohol consumption, restricting the analysis to individuals who had not altered their alcohol intake in the past five years. In supplementary analyses, we additionally adjusted for educational attainment (pre-secondary education, secondary education, or post-secondary education). However, as the findings remained virtually unchanged, this variable was not included in the final models to maintain parsimony and avoid over-adjustment. All analyses were conducted using Statistical Analysis System (SAS) version 9.4.

## Results

Our study included 3128 cases and 5986 controls. The mean age at onset was 34.5 years and the median duration from disease onset to the diagnosis was 1.0 year. Characteristics of cases and controls, in the overall sample and by a history of IM, are presented in Table 1.

**Table 1:** Characteristics of cases and controls, by IM status

	Prior infectious mononucleosis		No infectious mononucleosis	
	Cases	Controls	Cases	Controls
Total number	638	704	2490	5282
Mean age at index, *n* (%)	32.8 (9.3)	32.9 (9.6)	34.9 (10.9)	34.6 (11.0)
Women, *n* (%)	473 (74)	531 (75)	1768 (71)	3839 (73)
Nordic, *n* (%)	541 (85)	576 (82)	1983 (80)	4131 (78)
University studies, *n* (%)^1^	265 (53)	299 (51)	924 (45)	2032 (46)
Never smoking, *n* (%)	326 (51)	389 (55)	1162 (47)	3074 (58)
Current smoking, *n* (%)	173 (27)	159 (23)	749 (30)	1222 (23)
Past smoking, *n* (%)	138 (22)	156 (22)	578 (23)	985 (19)
Passive smoking, *n* (%)	330 (52)	306 (43)	1328 (53)	2481 (47)
Underweight, *n* (%)	72 (11.3)	80 (11.4)	299 (12.0)	727 (13.8)
Normal weight, *n* (%)	438 (68.7)	550 (78.1)	1749 (70.2)	3902 (73.9)
Overweight, *n* (%)	91 (14.3)	60 (8.5)	328 (13.2)	516 (9.8)
Obese, *n* (%)	37 (5.8)	14 (2.0)	114 (4.6)	137 (2.6)
Sun exposure index, mean (SD)	6.2 (1.7)	6.7 (2.0)	6.2 (1.9)	6.5 (1.9)
Sun exposure index, median (range)	6.0 (3–11)	7.0 (3–12)	6.0 (3–12)	6.0 (3–12)
Fish intake, mean (SD)	4.4 (1.1)	4.0 (1.2)	4.1 (1.1)	4.2 (1.1)
Fish intake, median (range)	4.0 (2–6)	4.0 (2–8)	4.0 (2–8)	4.0 (2–8)
Alcohol consumption (**♀**), mean (SD)	35 (50)	44 (60)	36 (65)	38 (60)
Alcohol consumption (**♀**), median (range)	24 (0-408)	29 (0-450)	18 (0-900)	24 (0-1250)
Alcohol consumption (**♂**), mean (SD)	69 (83)	82 (105)	62 (74)	85 (113)
Alcohol consumption (**♂**), median (range)	48 (0-655)	56 (0-900)	36 (0-600)	55 (0-1279)
Vitamin D level, mean (SD)	62 (25)	66 (25)	61 (27)	64 (26)
Vitamin D level, median (range)	59 (13–146)	68 (10–139)	59 (7-235)	62 (12–197)
Habitual sleep duration, mean (SD)	8.0 (1.0)	8.1 (1.1)	8.0 (1.1)	8.1 (1.0)
Habitual sleep duration, median (range)	8.0 (4–11)	8.0 (4–12)	8.0 (4–12)	8.0 (5–12)
Sleep quality, mean (SD)	4.2 (0.9)	4.3 (0.9)	4.3 (0.9)	4.4 (0.8)
Sleep quality, median (range)	4.0 (1–5)	5.0 (1–5)	5.0 (1–5)	5.0 (1–5)

^1^data on education was available for participants included before 2019 (2831 cases, 5446 controls)

In the overall sample, the OR of developing MS among those who reported a history of IM was 1.86 (95% CI 1.63–2.12), compared with those who had not suffered from IM. We observed synergistic effects between a history of IM and each exposure variable with respect to risk of MS, with significant APs ranging between 0.20 and 0.35 (Tables 2, 3, 4 and 5 and supplementary Tables 1–2).

**Table 2:** OR of MS with 95% CI for participants with different IM status and exposure to passive smoking

			Total sample		Never smokers
Cases/controls	IM	Passive smoking	OR (95% CI)^a^	OR (95% CI)^b^	OR (95% CI)^c^
1162/2801	-	-	1.0 (reference)	1.0 (reference)	1.0 (reference)
1328/2481	-	+	1.30 (1.18–1.43)	1.22 (1.11–1.35)	1.26 (1.10–1.45)
308/398	+	-	1.85 (1.57–2.18)	1.83 (1.55–2.15)	2.08 (1.68–2.58)
330/306	+	+	2.61 (2.20–3.09)	2.50 (2.10–2.96)	2.91 (2.29–3.71)
				AP 0.18 (0.01–0.35)	AP 0.20 (0.00-0.42)

^a^adjusted for age, sex, residential area, and ancestry; ^b^adjusted for age, sex, residential area, ancestry, smoking, sun exposure, and adolescent body mass index; ^c^adjusted for age, sex, residential area, ancestry, sun exposure, and adolescent body mass index; OR = odds ratio; CI = confidence interval; AP = attributable proportion due to interaction

### Passive smoking

Among participants without a history of IM, the OR of MS was 1.26 (95% CI 1.10–1.45) among those exposed to passive smoking, compared to non-exposed. However, the risk of MS was nearly tripled (OR 2.91, 95% CI 2.29–3.71) among the doubled-exposed (IM and passive smoking), compared to the reference group of non-exposed individuals without past IM (Table 2).

### Alcohol consumption

Low alcohol consumption rendered an OR of 1.48 (95% CI 1.33–1.67) among participants without a history of IM, whereas the risk of MS was tripled (OR 2.93, 95% CI 2.50–3.45) among doubled-exposed individuals (IM and low alcohol consumption). The AP was 0.20 (95% CI 0.04–0.38). Similar estimates were observed when the analysis was restricted to individuals who had not altered their alcohol intake in the past five years (AP 0.23, 95% CI 0.01–0.52) and when non-drinkers of alcohol were excluded (AP 0.19, 95% CI -0.07-0.46) (Table 3).

**Table 3:** OR of MS with 95% CI for participants with different IM status and alcohol consumption

Cases/controls	IM	Alcohol intake below median	OR (95% CI)^a^	OR (95% CI)^b^	AP (95% CI)
550/1455	-	-	1.0 (reference)	1.0 (reference)	
1940/3827	-	+	1.35 (1.21–1.51)	1.48 (1.33–1.67)	
153/220	+	-	1.85 (1.47–2.32)	1.83 (1.46–2.32)	
485/484	+	+	2.66 (2.27–3.12)	2.93 (2.50–3.45)	0.20 (0.04–0.38)

^a^adjusted for age, sex, residential area, and ancestry; ^b^adjusted for age, sex, residential area, ancestry, smoking, sun exposure, and adolescent body mass index; OR = odds ratio; CI = confidence interval; AP = attributable proportion due to interaction

### Fish consumption

Low consumption of fish among participants without past IM rendered a small increased risk of MS (OR 1.12, 95% CI 1.00-1.26), and interacted synergistically with a history of IM with respect to MS risk (AP 0.25, 95% CI 0.05–0.45) (Table 4).

**Table 4:** OR of MS with 95% CI for participants with different IM status and fish consumption

Cases/controls	IM	Fish intake below median	OR (95% CI)^a^	OR (95% CI)^b^	AP (95% CI)
811/1671	-	-	1.0 (reference)	1.0 (reference)	
1661/3590	-	+	1.13 (1.01–1.27)	1.12 (1.00-1.26)	
200/233	+	-	1.55 (1.20-2.00)	1.54 (1.19-2.00)	
431/468	+	+	2.23 (1.88–2.64)	2.23 (1.88–2.65)	0.25 (0.05–0.45)

^a^adjusted for age, sex, residential area, and ancestry; ^b^adjusted for age, sex, residential area, ancestry, smoking, sun exposure, and adolescent body mass index; OR = odds ratio; CI = confidence interval; AP = attributable proportion due to interaction

### Vitamin D

Low vitamin D levels among those without IM rendered an OR of 1.19 (95% CI 1.00-1.40) whereas double-exposed individuals (IM in combination with low vitamin D) had an OR of MS of 2.69 (95% CI 1.89–3.82), rendering an AP of 0.35 (0.06–0.64) (Table 5).

**Table 5:** OR of MS with 95% CI for participants with different IM status and vitamin D status

Cases/controls	IM	Vitamin D level below median	OR (95% CI)^a^	OR (95% CI)^b^	AP (95% CI)
446/606	-	-	1.0 (reference)	1.0 (reference)	
562/636	-	+	1.22 (1.03–1.44)	1.19 (1.00-1.40)	
90/76	+	-	1.56 (1.12–2.17)	1.56 (1.12–2.18)	
109/54	+	+	2.69 (1.90–3.82)	2.69 (1.89–3.82)	0.35 (0.06–0.64)

^a^adjusted for age, sex, residential area, and ancestry; ^b^adjusted for age, sex, residential area, ancestry, month of blood sampling, smoking, sun exposure, and adolescent body mass index; OR = odds ratio; CI = confidence interval; AP = attributable proportion due to interaction

### Sleep variables

Both sleep duration and low sleep quality interacted synergistically with a history of IM, rendering APs of 0.30 (95% CI 0.05–0.56) and 0.29 (95% CI 0.00-0.66), respectively (Tables 6 and 7).

**Table 6:** OR of MS with 95% CI for participants with different IM status and habitual sleep duration (age 15–19 years)

Cases/controls	IM	Habitual sleep duration < median	OR (95% CI)^a^	OR (95% CI)^b^	AP (95% CI)
654/1132	-	-	1.0 (reference)	1.0 (reference)	
863/1460	-	+	0.98 (0.84–1.13)	1.01 (0.87–1.18)	
146/155	+	-	1.49 (1.12-2.00)	1.49 (1.12–1.99)	
240/185	+	+	2.10 (1.63–2.72)	2.14 (1.66–2.76)	0.30 (0.05–0.56)

^a^adjusted for age, sex, residential area, and ancestry; ^b^adjusted for age, sex, residential area, ancestry, month of blood sampling, smoking, sun exposure, and adolescent body mass index; OR = odds ratio; CI = confidence interval; AP = attributable proportion due to interaction

**Table 7:** OR of MS with 95% CI for participants with different IM status and sleep quality (age 15–19 years)

Cases/controls	IM	Sleep quality < median	OR (95% CI)^a^	OR (95% CI)^b^	AP (95% CI)
1282/2282	-	-	1.0 (reference)	1.0 (reference)	
235/310	-	+	1.31 (1.05–1.64)	1.31 (1.05–1.64)	
315/297	+	-	1.78 (1.45–2.18)	1.78 (1.45–2.18)	
71/43	+	+	2.95 (1.81–4.79)	2.95 (1.81–4.79)	0.29 (0.00-0.66)

^a^adjusted for age, sex, residential area, and ancestry; ^b^adjusted for age, sex, residential area, ancestry, month of blood sampling, smoking, sun exposure, and adolescent body mass index; OR = odds ratio; CI = confidence interval; AP = attributable proportion due to interaction

## Discussion

The concept of EBV infection as a crucial factor for MS gains further support from our findings suggesting that other MS risk factors synergize with a history of IM in disease development. It is conceivable that inadequate control of this infection represents another prerequisite for disease onset. Viral reactivation can be triggered by various stressors, whether immune, cellular, or psychological. Targeting modifiable MS risk factors that contribute to inadequate viral control through preventive measures could therefore be effective.

In addition to the observed interactions between various aspects of EBV and MS risk factors such as HLA genotype [6–8], smoking (9–10), obesity [11] and low sun exposure [12], our study identified additive interactions between a history of IM and each of the variables examined (passive smoking, alcohol consumption, fish consumption, vitamin D, and sleep habits) in relation to MS risk.

Similar to the observed interaction between smoking and aspects of EBV infection (9–10), exposure to tobacco smoke among never smokers acted synergistically with a history of IM to increase the risk of MS. Besides inducing proinflammatory responses (15–16), smoke exposure results in a relative immune deficiency [16]. Studies have linked smoking to frequent EBV reactivation, resulting in elevated EBV antibodies against viral antigens (17–18). Experimental evidence supports this association, demonstrating EBV replication and expression of lytic-phase genes following exposure to cigarette smoke [19]. Smoking also induces alterations in the distribution and function of memory B cells, leading to a higher prevalence of these cells in peripheral blood and lung tissues [20]. Furthermore, smoking-related pulmonary inflammation may activate tissue-resident immune cells within the lung, potentially including autoreactive effector and memory cells, and facilitate their migration into the CNS [21].

Active vitamin D regulates the expression of over 200 genes and plays a multifaceted role in modulating the immune system. It enhances the production of antimicrobial peptides and NK cells, which are crucial for combating viruses (22–23). Studies on patients with MS have revealed an inverse correlation between serum vitamin D levels and EBNA-1 antibody levels (24–25), suggesting that vitamin D deficiency might compromise the T cell-mediated response to EBV, potentially leading to viral reactivation and increased antibody production. Some evidence suggest that vitamin D levels could influence the interplay between EBV and genetic predispositions for MS. EBNA2, a key transcriptional activator involved in B cell transformation, binds to MS susceptibility loci within the host genome of infected B cells, potentially affecting their gene expression [26]. The observed overlap between EBNA2 and VDR binding sites, indicates a possible competition between EBNA2 and vitamin D receptor (VDR) for binding to these susceptibility loci, many of which are associated with immune responses [27]. Given the potentially antagonistic effects of EBV and vitamin D on B cell function, maintaining adequate levels of vitamin D may confer protection against MS by limiting EBNA2 genomic occupancy and its influence on B cell activity.

The compromised ability to manage viral infections due to insufficient sleep may contribute to explain the association between inadequate sleep and increased risk of MS [28]. Alcohol and polyunsaturated fatty acids possess immunomodulatory properties (29–30), yet the precise mechanisms through which they influence MS risk remain unclear. While studies on dietary habits, including alcohol intake, should be interpreted with caution due to potential sources of bias, their interactions with a history of IM in MS development support their involvement in MS etiology.

While numerous environmental factors and genetic variants predispose to MS, their biological significance largely remains uncertain, often with low or modest effects. Some environmental factors may have multiple significant biological effects in pathogenesis, while others impact a small set of shared pathways, resulting in similar biological effects. While the ideal preventive measure would be EBV vaccination, targeting modifiable MS risk factors that contribute to inadequate viral control through preventive measures could be effective.

Based on the presented and previous findings, one strategy could involve balanced information about the risks and benefits of sun exposure. The ultraviolet index can be useful for guiding sun exposure behavior in accordance with WHO guidelines to minimize the risk of skin cancer [31], but sensible sun exposure is essential for vitamin D synthesis and for capturing other vitamin D-independent benefits. Vitamin D supplementation may also be considered as part of preventive strategies, particularly for individuals with limited sun exposure or those living at higher latitudes. Both sun exposure and vitamin D supplementation may act through overlapping and independent pathways to influence immune regulation in MS. Addressing the prevalence of smoking requires a multi-faceted approach, including governmental regulations, school-based prevention programs, cessation supports for smokers, and improved access to cessation resources. Educating parents about the importance of protecting their children from secondhand smoke is crucial. Childhood obesity is a significant concern, necessitating promotion of healthy eating, regular physical activity, limited screen time, and good sleep habits. Healthcare professionals can provide guidance on nutrition and physical activity strategies for at-risk children. The health benefits of Mediterranean-style diet for obesity-related conditions are well-documented^32^, emphasizing high consumption of vegetables, fruits, nuts, fish, and olive oil. Promoting healthy sleep patterns is vital for overall immune health. Educational initiatives targeting adolescents and parents, highlighting the adverse health effects of insufficient sleep, are important. These guidelines align with those for general health benefits and may have the potential to lower the risk of MS. Conversely, incorporating findings on the potential benefits of alcohol in MS presents challenges due to the well-documented adverse effects of alcohol consumption on human health. Nevertheless, further investigation into underlying mechanisms may provide insights into alternative interventions for MS risk reduction that do not involve alcohol.

Using a history of IM as a proxy for EBV control has important implications for interpreting our findings. IM, as a clinical manifestation of primary EBV infection, may reflect inadequate immune control of the virus [4]. This suggests that individuals who experience IM could have an immune response to EBV that is less effective in controlling viral latency or reactivation, potentially increasing their risk of MS.

However, it is important to note that not all cases of IM are clinically recognized or reported, which could lead to misclassification of EBV control in some participants. Furthermore, IM may not fully capture the complexity of host-EBV interactions, as factors such as genetic predisposition, immune function, and environmental exposures also influence viral control. Despite these limitations, the strong association between IM and MS risk supports its utility as a proxy for investigating EBV-related mechanisms in MS pathogenesis.

Our study employed a population-based case-control design, with retrospective collection of exposure data. Case-control studies are inherently susceptible to certain biases, including recall and selection bias. To mitigate potential recall bias, we focused on including cases diagnosed within the past year. However, we acknowledge that the risk of recall bias remains, as participants may still inaccurately recall past exposures. Another potential concern is that the recruitment of cases and controls may have introduced selection bias. However, the Swedish healthcare system offers free medical services to all citizens, likely capturing almost all MS cases. Therefore, the likelihood of unidentified MS cases biasing our findings is low. However, we recognize that the 27% non-response rate among controls introduces the potential for selection bias. There were no significant differences in demographic or lifestyle factors between participants who completed supplementary sleep questions and those who did not, suggesting minimal selection bias in that step. Similarly, no significant differences were observed between participants who provided blood samples and those who did not, indicating no bias in this step either. Unmeasured confounders or imperfectly measured variables may still have influenced our findings. Nonetheless, the consistency of our results across multiple sensitivity analyses strengthens their validity.

In conclusion, our findings support the notion of EBV playing a crucial role in MS, as evidenced by the synergistic interactions observed between EBV and various MS risk factors. It is conceivable that insufficient control of the EBV infection represents another prerequisite for disease onset. Viral reactivation may be precipitated by a myriad of stressors, spanning immune, cellular, and psychological domains. Targeting modifiable MS risk factors that impede effective immune regulation of the virus holds promise for preventive interventions.

## Data Availability

Anonymized data will be shared by request from any qualified investigator that wants to analyse questions that are related to the published article.

## References

[1] Bjornevik K, Cortese M, Healy BC, Kuhle J, Mina MJ, Leng Y, et al. Longitudinal analysis reveals high prevalence of Epstein-Barr virus associated with multiple sclerosis. Science. 2022;375:296–301. 35025605 10.1126/science.abj8222

[2] Pakpoor J, Disanto G, Gerber JE, Dobson R, Meier UC, Giovannoni G, et al. The risk of developing multiple sclerosis in individuals seronegative for Epstein-Barr virus: a meta-analysis. Mult Scler. 2013;19:162–6. 22740437 10.1177/1352458512449682

[3] Rothman KJ, Greenland S, Lash TL. Modern Epidemiology, 3rd edition, Philadelphia: Lippincott Wolliams & Wilkins (2008).

[4] Hedström AK. Risk factors for multiple sclerosis in the context of Epstein-Barr virus infection. Front Immunol. 2023;14:1212676. 37554326 10.3389/fimmu.2023.1212676 PMC10406387

[5] Handel AE, Williamson AJ, Disanto G, Handunnetthi L, Giovannoni G, Ramagopalan SV. An updated meta-analysis of risk of multiple sclerosis following infectious mononucleosis. PLoS ONE. 2010;5:e12496. 20824132 10.1371/journal.pone.0012496 PMC2931696

[6] Disanto G, Hall C, Lucas R, Ponsonby AL, Berlanga-Taylor AJ, Giovannoni G, et al. Assessing interactions between HLA-DRB1*15 and infectious mononucleosis on the risk of multiple sclerosis. Mult Scler. 2013;19:1355–8. 23413297 10.1177/1352458513477231

[7] De Jager PL, Simon KC, Munger KL, Rioux JD, Hafler DA, Ascherio A. Integrating risk factors: HLA-DRB1*1501 and Epstein-Barr virus in multiple sclerosis. Neurology. 2008;70:1113–8. 18272866 10.1212/01.wnl.0000294325.63006.f8

[8] Hedström AK, Hillert J, Brenner N, Butt J, Waterboer T, Strid P, et al. DRB1-environment interactions in multiple sclerosis etiology: results from two Swedish case-control studies. J Neurol Neurosurg Psychiatry. 2021;92:717–22. 33687974 10.1136/jnnp-2020-325676 PMC8223646

[9] Salzer J, Stenlund H, Sundström P. The interaction between smoking and Epstein-Barr virus as multiple sclerosis risk factors may depend on age. Mult Scler (, Huang J, Brenner N, Butt J, Hillert J, Waterboer T et al. Smoking and Epstein-Barr virus infection in multiple sclerosis development. Sci Rep (2020) 10:10960.

[10] Hedström AK, Bomfim IL, Hillert J, Olsson T, Alfredsson L. Obesity interacts with infectious mononucleosis in risk of multiple sclerosis. Eur J Neurol. 2015;22:578–e38. 25530445 10.1111/ene.12620 PMC4365756

[11] Hedström AK, Huang J, Brenner N, Butt J, Kockum I, Waterboer T, et al. Low sun exposure acts synergistically with high Epstein-Barr nuclear antigen 1 (EBNA-1) antibody levels in multiple sclerosis etiology. Eur J Neurol. 2021;28:4146–52. 34435414 10.1111/ene.15082

[12] Polman CH, Reingold SC, Edan G, et al. Diagnostic criteria for multiple sclerosis: 2005 revisions to the McDonald criteria. Ann Neurol. 2005;58:840–6. 16283615 10.1002/ana.20703

[13] Polman CH, Reingold SC, Banwell B, et al. Diagnostic criteria for multiple sclerosis: 2010 revisions to the McDonald criteria. Ann Neurol. 2011;69:292–302. 21387374 10.1002/ana.22366 PMC3084507

[14] Sopori M. Effects of cigarette smoke on the immune system. Nat Rev Immunol. 2002;2:372–7. 10.1038/nri803. 12033743 10.1038/nri803

[15] Arnson Y, Shoenfeld Y, Amital H. Effects of tobacco smoke on immunity, inflammation and autoimmunity. J Autoimmun. 2010;34:J258–265. 20042314 10.1016/j.jaut.2009.12.003

[16] He Y, Liao X, Xue W, Xu YF, Xu FH, Li FF, et al. Association between environmental factors and oral Epstein-Barr virus DNA loads: a multicenter cross-sectional study in China. J Infect Dis. 2019;219:400–9. 30307559 10.1093/infdis/jiy542 PMC6941616

[17] Hu T, Lin C, Xie S, Chen GH, Lu YQ, Ling W, et al. Smoking can increase nasopharyngeal carcinoma risk by repeatedly reactivation Epstein-Barr virus: an analysis of a prospective study in Southern China. Cancer Med. 2019;8:2561–71. 30843658 10.1002/cam4.2083 PMC6536979

[18] Xu FH, Xiong D, Xu YF, Cao SM, Xue WQ, Qin HD, et al. An epidemiological and molecular study of the relationship between smoking, risk of nasopharyngeal carcinoma, and Epstein-Barr virus activation. J Natl Cancer Inst. 2012;104:1396–410. 22972969 10.1093/jnci/djs320

[19] Brandsma CA, Hylkema MN, Geerlings M, van Geffen WH, Postma DS, Timens W, et al. Increased levels of (class switched) memory B cells in peripheral blood of current smokers. Respir Res. 2009;10:108. 19909533 10.1186/1465-9921-10-108 PMC2779187

[20] Odoardi F, Sie C, Streyl K, Ulaganathan VK, Schläger C, Lodygin D, et al. T cells become licensed in the lung to enter the central nervous system. Nature. 2012;488:675–9. 22914092 10.1038/nature11337

[21] Waddell A, Zhao J, Cantorna MT. NKT cells can help mediate the protective effects of 1,25-dihydroxyvitamin D3 in experimental autoimmune encephalomyelitis in mice. Int Immunol. 2015;27:237–44. 10.1093/intimm/dxu147. 25574039 10.1093/intimm/dxu147 PMC4406266

[22] Gombart AF, Borregaard N, Koeffler HP. Human Cathelicidin antimicrobial peptide (CAMP) gene is a direct target of the vitamin D receptor and i strongly up-regulated in myeloid cells by 1,25-dihydroxyvitamin D2. FASEB J. 2005;19:1067–77. 15985530 10.1096/fj.04-3284com

[23] Salzer J, Nyström M, Hallmans G, Stenlund H, Wadell G, Sundström P. Epstein-Barr virus antibodies and vitamin D in prospective multiple sclerosis biobank samples. Mult Scler. 2013;19:1587–91. 23549431 10.1177/1352458513483888

[24] Wergeland S, Myhr KM, Løken-Armsrud KI, Beiske AG, Bjerve S, Hovdal H, et al. Vitamin D, HLA-DRB1 and Epstein–Barr virus antibody levels in a prospective cohort of multiple sclerosis patients. Eur J Neurol. 2016;23:1064–70. 26998820 10.1111/ene.12986

[25] Harley JB, Chen XX, Pujato M, Miller D, Maddox A, Forney C, et al. Transcription factors operate across disease loci, with EBNA2 implicated in autoimmunity. Nat Genet. 2018;50:699–707. 29662164 10.1038/s41588-018-0102-3 PMC6022759

[26] Ricigliano VAG, Handel AE, Sandve GK, Annibali V, Ristori G, Mechelli R, et al. EBNA2 binds to genomic intervals associated with multiple sclerosis and overlaps with vitamin D receptor occupancy. PLoS ONE. 2015;10:e0119605. 25853421 10.1371/journal.pone.0119605 PMC4390304

[27] Åkerstedt T, Olsson T, Alfredsson L, Hedström AK. Insufficient sleep during adolescence and risk of multiple sclerosis: results from a Swedish case-control study. J Neurol Neurosurg Psychiatry. 2023;94:331–6. 36690431 10.1136/jnnp-2022-330123 PMC10176406

[28] Romeo J, Wärnberg J, Nova E, et al. Moderate alcohol consumption and the immune system. Br J Nutr. 2007;98:S111–5. 17922947 10.1017/S0007114507838049

[29] Calder PC. Long chain fatty acids and gene expression in inflammation and immunity. Curr Opin Clin Nutr Metab Care. 2013;16:425–33. 23657154 10.1097/MCO.0b013e3283620616

[30] Gies P, van Deventer E, Green AC, Sinclair C, Tinker R. Review of the global solar UV index 2015 workshop report. Health Phys. 2018;114:84–90. 30085971 10.1097/HP.0000000000000742 PMC5728586

[31] Abrignani V, Salvo A, Pacinella G, et al. The mediterranean diet, its Microbiome connections, and cardiovascular health: a narrative review. Int J Mol Sci. 2024;25:4942. 38732161 10.3390/ijms25094942 PMC11084172

